# Effectiveness of lumbar stabilization exercise with real-time ultrasound imaging biofeedback on lumbar multifidus muscle cross-sectional area in individuals with non-specific chronic low back pain: a study protocol for a randomized controlled trial

**DOI:** 10.1186/s13063-021-05952-9

**Published:** 2022-01-06

**Authors:** Raheem Sarafadeen, Sokunbi O. Ganiyu, Aminu A. Ibrahim, Anas Ismail, Mukadas O. Akindele, Bashir Kaka, Adedapo W. Awotidebe

**Affiliations:** 1 Department of Physiotherapy, Faculty of Allied Health Sciences, College of Health Sciences, Bayero University, Kano, Kano State P.M.B 3011 Nigeria; 2 Department of Physiotherapy, National Orthopedic Hospital, Dala, Kano, Kano State P.M.B 3087 Nigeria; 3 Department of Physiotherapy, Muhammad Abdullahi Wase Teaching Hospital, Hospitals Management Board, Kano, Kano State P.M.B 3160 Nigeria; 4 Department of Radiology, Aminu Kano Teaching Hospital, Kano, Kano State P.M.B 3452 Nigeria

**Keywords:** Chronic low back pain, Lumbar stabilization exercise, Lumbar multifidus muscle, Cross-sectional area, Pain, Functional disability, Real-time ultrasound imaging biofeedback, Quality of life

## Abstract

**Background:**

Structural impairment of the lumbar multifidus muscle, such as reduced cross-sectional area, is evident among individuals with chronic low back pain. Real-time ultrasound imaging (RUSI) biofeedback has been reported to improve preferential activation of as well as retention in the ability to activate the lumbar multifidus muscle during lumbar stabilization exercises (LSE). However, evidence of the effectiveness of this treatment approach in individuals with non-specific chronic low back pain (NCLBP) is still limited. The purpose of this study is, therefore, to determine the effectiveness of LSE with RUSI biofeedback on lumbar multifidus muscle cross-sectional area in individuals with NCLBP.

**Methods/Design:**

This study is a prospective, single-center, assessor-blind, three-arm, parallel randomized controlled trial to be conducted at National Orthopedic Hospital, Kano State, Nigeria. Ninety individuals with NCLBP will be randomized in a 1:1:1: ratio to receive LSE, LSE with RUSI biofeedback, or minimal intervention. All participants will receive treatment twice weekly for 8 weeks. The primary outcome will be the lumbar multifidus muscle cross-sectional area. The secondary outcomes will include pain (Numerical Pain Rating Scale), functional disability (Roland–Morris Disability Questionnaire), and quality of life (12-Item Short-Form Health Survey). All outcomes will be assessed at baseline, 8 weeks post-intervention,  and 3 months follow-up.

**Discussion:**

To our knowledge, this study will be the first powered randomized controlled trial to compare the effectiveness of LSE training with and without RUSI biofeedback in individuals with NCLBP. The outcome of the study may provide evidence for the effectiveness of LSE with RUSI biofeedback on enhancing the recovery of the lumbar multifidus muscle in individuals with NCLBP.

**Trial registration:**

Pan African Clinical Trials Registry (PACTR201801002980602). Registered on January 16, 2018.

**Supplementary Information:**

The online version contains supplementary material available at 10.1186/s13063-021-05952-9.

## Background

Low back pain (LBP) is a common musculoskeletal disorder causing years lived with disability than any other condition around the world [1]. It is a major public health issue owing to its multifactorial impact including pain, activity limitations, participation restrictions, career burden, healthcare utilization, and huge economic costs [2, 3]. Moreover, the burden of LBP is projected to increase over the coming decades due to population increase and aging [3]. Hence, there is a need to prioritize LBP and identify effective intervention strategies to reduce the consequences of the current and projected future burden 3.

Although many sp inal structures could be claimed responsible for the origin of LBP, for up to 9 5% of cases, the specific cause of the pain cannot be reliably identified [4]. However, prior biome chanical studies suggest that impairments in the key stabilizing muscles of the spine could be attributed to the development or recurrence of subacute and non-specific chronic back complaints [5]. For example, it has been documented that structural impairments of the lumbar multifidus muscles (LMM), such as reduced cross-sectional area (CSA) [6–9], thickness [10–12], and increased fat infiltrations [7, 9, 13–15], are evident among individuals with chronic LBP. 

One treatment approach believed to be able to improve these LMM dege nerative changes is lumbar stabilization exercise (LSE) program. This specific exercise approach is commonly applied by physiotherapists to rehabilitate individuals with chronic low back disorders [16–20]. Importantly, there is evidence to support its efficacy in improving pain, functional disabil ity, and quality of life (QOL) among sufferers of chronic LBP even though it may not be superior to other types of exercise programs [21, 22].

The focus of LSE is to train the skilled activation of the deep trunk muscles particularly the LMM and transversus abdominis (TrA) muscle [23]. Improving the activation capacity of the LMM is believed to enhance its function (i.e., providing segmental stability and controlling intervertebral motion [24]) by reversing the degenerative changes commonly seen in the muscle [5]. However, to improve the precision of muscle contraction while performing LSE, it is crucial to provide accurate feedback during the training. This may involve any of the senses including tactile (palpation), auditory (electr omyography), and visual ultrasound imaging information [25]. Though the use of palpatory feedback is commonly practiced, v isual feedback using real-time ultrasound imaging (RUSI) has been shown to be promising in providing preferential activation of the deep trunk musculature during LSE in asymptomatic individuals [26, 27]. More specifically, Van et al. [27] asserted that the use of RUSI biofeedback may improve not only LMM activation performance but also retention in the ability to activate the muscle, which is crucial for individuals with LBP as reoccurrences are common [18]. Consequently, the  use of this bio feedback approach may reduce the number of trials required to consistently perform isometric contraction to activate the deep stabilizing trunk muscles [26].

Most trials examining the effectiveness of LSE on LMM CSA or thickness did not provide RUSI as biofeedback to improve LMM preferential activation or contraction quality. Although previous but small trials found LSE with RUSI to be effective at improving LMM CSA [28–30], pain, and disability [28, 29] in patients with non-specific LBP, evidence of the effectiveness of this treatment approach among patients with non-specific chronic LBP (NCLBP) is still limited due to lack of rigorous trials in the way of randomized controlled trials (RCTs). Of the four trials examining the effectiveness of LSE with RUSI on LMM CSA [28–30] or thickness [31] in patients with non-specific acute LBP [28] or NCLBP [29–31], all suffered a small sample size (*n* = 10–41) without a priori power calculation, and two were non-RCT [29, 30] of which one included  elite cricketers [30].

According to the latest systematic review [32] to summarize the evidence regarding the effectiveness of LSE on LMM morphology, very low- to low-quality evidence exists that LSE improves LMM CSA post-intervention or compared to medication, general exercise, or general physiotherapy in patients with non-specific LBP. However, only one RCT [28] (conducted among acute LBP patients) of the five included RCTs [28, 33–36] used RUSI as biofeedback. Furthermore, on the basis of two small trials [28, 37] in which one included patients with acute LBP [28] and the other included patients with  chronic LBP [37], the review [32] found no evidence to support the correlation between changes in LMM CSA  and changes in pain or disability. Thus, it is imperative to explore  if the addition of RUSI biofeedback in LSE training would provide more favorable effect on LMM CSA compared to LSE training without RUSI biofeedback, and to examine the correlation between changes in LMM CSA and LBP or LBP-related disability among individuals with NCLBP. 

Our recent pilot study [29], even though was limited to being a single-group pretest-posttest design, suggests the feasibility of conducting a large-scale RCT  to determine the efficacy of LSE with RUSI biofeedback in individuals with NCLBP. The use of LSE with RUSI biofeedback resulted in significant improvement in LMM CSA by 1.7 points, pain by 3.9 points, and  disability by  4.5 points. However, no significant improvement in  physical and mental health was observed after the intervention, which could be attributed to the shorter treatment (12 sessions) provided [29]. Thus, the purpose of this study is to determine the effectiveness of LSE with RUSI biofeedback in individuals with NCLBP. The primary outcome will be LMM CSA whereas the secondary outcomes include  pain, functional disability, and QOL. 

## Methods/Design

### Hypothesis 

We hypothesized that patients receiving LSE with RUSI biofeedback will demonstrate significant improvement in LMM CSA, pain, functional disability, and QOL compared to those receiving LSE without RUSI biofeedback or minimal intervention (control). 

### Primary objective

To determine the effectiveness of LSE with RUSI biofeedback on LMM CSA in individuals with NCLBP.

### Secondary objectives 

To determine the effec tiveness of LSE with RUSI biofeedback on pain, functional disability, and QOL in individuals with NCLBP. Additionally, we will determine the association between  post-treatment scores in LMM CSA and post-treatment scores in pain and functional disability.  

### Study design 

**Fig. 1:**
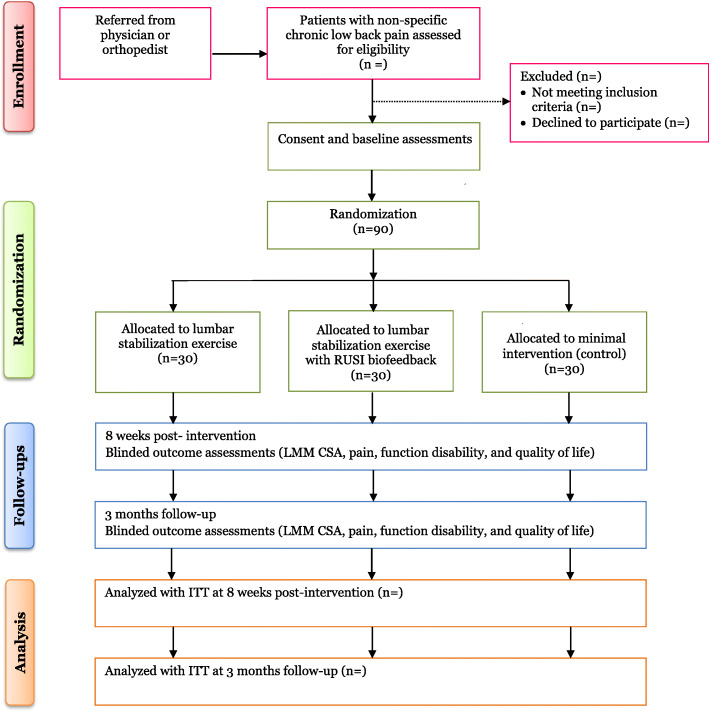
Flow of participants through the study

This study will be a prospective, single-center, assessor-blind, three-arm, parallel RCT and will be conducted at the Physiotherapy Department, National Orthopedic Hospital, Dala, Kano State, Nigeria. The outline of the study is presented in Fig. 1. The protocol for this study is reported in accordance with the Standard Protocol Items: Recommendations for Interventional Trials (SPIRIT) 2013 Checklist (Additional file 1).

### Physiotherapists/research assistants

The principal investigator (RS) and two physiotherapists (with about 4 to 6 years of working experience in musculoskeletal physiotherapy)  will be responsible for patients’ screening, appointment, and treatment. A consultant radiologist (with over 10 years of experience) blinded to group allocation in diagnostic ultrasound imaging will be responsible for ultrasound imaging and measurements. Another physiotherapist (with about 3 years of working experience in musculoskeletal physiotherapy) blinded to group allocation will be responsible for the assessment of self-report questionnaires. All the physiotherapists will receive a training session and written instructions for the study protocols.

### Participants and recruitment

Patients with LBP attending outpatient physiotherapy and those referred for physiotherapy by orthopedists will be screened for their suitability to participate in the study. Participants’ eligibility will be ensured through history taking and physical examinations. The participants will be included if they (1) are male or female between the age of 18 and 60 years, (2) have a primary complaint of non-specific LBP with or without leg pain for 12 weeks or more, and (3) are able to read and understand Hausa or English language or both. Part icipants will be excluded if they (1) have a history of spine surgery; (2) have obvious deformities affecting the trunk or upper and lower extremities; (3) have serious spinal pathology such as infection, fracture, metastases, and cauda equine syndrome; (4) have a history of medical or surgical conditions that might hinder exercise performance; and (5) are pregnant or lactating women. Participants meeting the eligibility criteria and who accept willingly to participate will be given oral and written information about the study procedures. They will also be informed about their rights to withdraw from participation at any time without prejudice. Informed consent will be obtained via signature or thumbprint.  Participants’ baseline demographic and clinical variables, such as age, gender, marital status, education level, employment status, pain duration, height, weight, and body mass index will be collected and recorded after group allocation. The participants will be identified only by their initials using  research notes.

### Rando mization and blinding

A record clerk who will not be involved in the assessment and treatment of participa nts will be responsible for the random allocation of participants into different intervention groups. The participants will be randomly assigned into LSE group, LSE with RUSI biofeedback (LSER) group,  or minimal in tervention (control) group in a 1:1:1 ratio (i.e., 30 participants per group) (Fig. 1). Consecu tively numbered (1 to  90) sealed opaque envelopes will be prepared using a computer-generated randomization sequence with permuted blocks of varying sizes (i.e., 3 and  6) by an independent statistician. Once a participant fulfilled the inclusion criteria and give consent for the study, the record clerk will perform the randomization by picking a sequential envelope and assign the participant to the group written in the envelope. All assessors will be blinded to participants’ group allocation. However, because of the nature of the interventions, it will be difficult to blind the participants and investigators (physiotherapists) to treatment allocation. Unbinding conditions will not occur but maybe only permissible when there is a medical emergency.

### Outcome assessment

Primary outcome will be LMM CSA assessed with RUSI whereas secondary outcomes will include pain, functional disability, and QOL to be assessed using self-report questionnaires. All outcomes will be assessed at baseline (−*t*_*1*_), 8 weeks post-intervention (*t*_*2*_), and 3 months follow-up (*t*_*3*_) (Table 1).

**Table 1:**
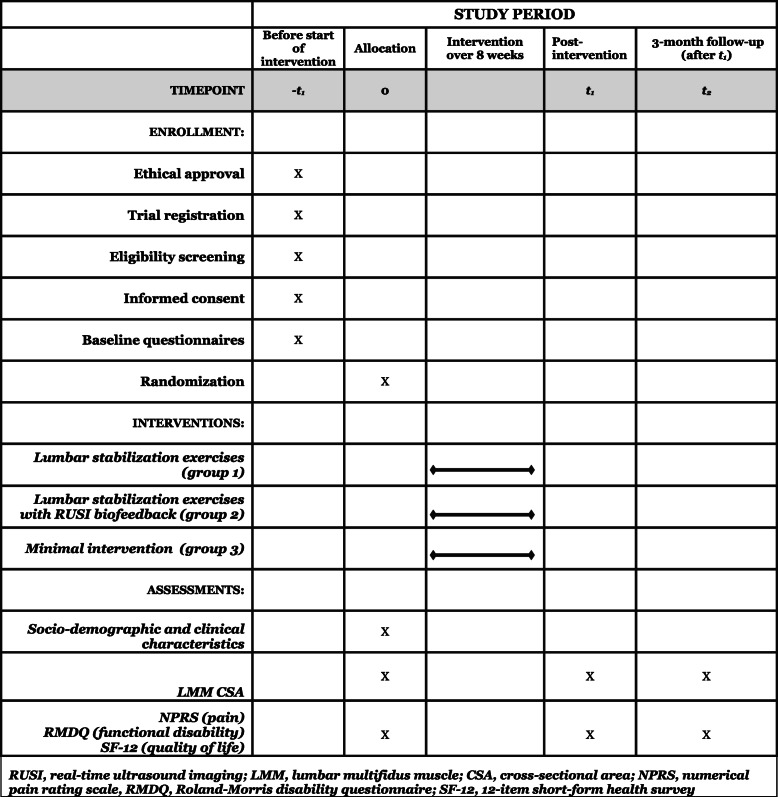
SPIRIT figure: time points for en rollment, interventions, and assessment

#### Lumbar multifidus muscle cross-sectional area

The procedure for the LMM CSA will be identical to that described in previous studies [30, 38]. We found a within-day assessor intra-rater reliability during our pilot trial (*n* = 10) [29] to be excellent (intraclass correlation coefficients = 0.96) similar to findings of previous studies [39, 40]. Also, LMM CSA measurements obtained using RUSI have been validated by comparison with magnetic resonance imaging measurements [40]. The imaging of the LMM CSA will be measured at the L5 level using real-time ultrasound apparatus (Edan D3 version 1.6, China) with a 5-MHz curvilinear transducer. Participants will be placed on prone lying, on a plinth with arms by their sides and head turned to a preferred side. To keep the lumbar spine in a neutral position, a pillow will be placed under the hips to make the lower lumbar spine flat. The L5 spinous process will be identified by palpation, and the skin will be marked using a pen for reference. An acoustic coupling gel will be applied to the skin and the transducer head. Thereafter, the transducer head will be placed longitudinally along the midline of the lumbar spine to confirm the L5 level. The transducer head will be then placed transversely over the spinous process of L5 to obtain imaging of the spinous process and laminae along with the LMM on both sides of the spine. Transverse images of the LMM will be obtained bilaterally with the small side (thinner) being considered as the LMM CSA of the patient. Measurements will be taken during maximal voluntary isometric contraction. To ensure accuracy, average measures of three images of the LMM will be taken. The L5 level is chosen in this study because previous studies indicate that decreased LMM CSA and increased side-to-side asymmetry are common at this level [30, 41].

#### Pain

The level of pain intensity of the participants will be assessed by administering the Numerical Pain Rating Scale (NPRS). The NPRS consists of an 11-point Likert scale, with 0 representing “no pain” and 10 representing “worst imaginable pain". The participants will be asked to indicate the best point that represents the greatest pain they experienced at the time of assessment. Both the Hausa [42] and English [43] versions of the NPRS will be used in the present study. The NPRS has been reported to be a valid, reliable, and responsive measure of pain intensity in patients with LBP [42–44].

#### Functional disability

The levels of functional disability of the participants will be assessed by administering the Roland–Morris Disability Questionnaire (RMDQ). It is a 24-item questionnaire, with scores ranging from 0 (no disability) to 24 (maximum disability). The participants will be asked to tick on any of the 24 items that describe their current disability level. To obtain the RMDQ total score, the items checked are summed up. Both the Hausa [45] and English [46] versions of the RMDQ will be used in the present study. The questionnaire has been reported to be a reliable and valid measure of LBP-related disability [45, 46].

#### Quality of life

The QOL (physical and mental health) of the participants will be assessed by administering the 12-item Short-Form Health Survey (SF-12) questionnaire. The SF-12 is a shorter alternative to the SF-36 Health Survey. The questionnaire measures two health constructs— physical component scores (PCS-12) and mental component scores (MCS-12). A Web-based scoring tool (www.orthotoolkit.com/sf-12/) will be used to calculate the PCS-12 and MCS-12 scores, with higher scores indicating better health status. Both the Hausa [47] and English [48] versions of the SF-12 will be used in the present study. The questionnaire has been reported to be reliable and valid for measuring health-related quality of life [47, 48].

### Interventions

All interventions will be provided twice a week for 8 weeks and under the supervision of the physiotherapists. Participants in the LSE and LSER groups will also carry out the same intervention as for the control group (minimal intervention). All participants will be advised not to partake in any exercise-related interventions during the study. To enhance retention, participants will be contacted by the research coordinator via text messages, WhatsApp messenger, and phone calls on a regular basis to remind them of their treatment appointments  and post-treatment follow-up.

#### Lumbar stabilization exercise (LSE)

Participants allocated to this group will be taught LSE based on the approach of Richardson and colleagues [23] and procedures explained in previous studies [29, 30, 40]. Prior to the training, the participants will be educated with aid of video and pictures on the anatomical location and function of the lumbar spine and the key deep stabilizing muscles with a focus on LMM. They will be asked to assume a prone lying on a treatment table with a pillow placed under the hips to ensure neutral positioning of the spine. Participants will be taught an active isometric contraction of the LMM (with a focus on L2–L5 vertebral level by drawing up the anterior aspect of the pelvic floor or lifting the coccyx to ceiling) in synergy with other deep stabilizing muscles such as TrA (abdominal draw-in maneuver) [30]. Contractions will be held for 10 s and repeated 10 times, with a period of 30- to 60-s rest between repetitions.

#### Lumbar stabilization exercise with real-time ultrasound imaging biofeedback (LSER)

Herein, the intervention will be similar to that described in the LSE group except for the addition of RUSI biofeedback to enhance the precision of contraction and activation performance of the LMM. While maintaining a neutral spine in prone lying, the transducer head of the ultrasound will be placed transversely over the L2–L5 levels and then the participants will be instructed to perform isometric contraction of the LMM and other deep stability muscles as described above. Specifically, they will be instructed to focus on the monitor to see the changes in the thickness of the LMM as they contract the muscles and put in their best effort to increase the thickness with successive contractions. Contractions will be held for 10 s and repeated 10 times, with a period of 30- to 60-s rest between repetitions.

#### Minimal intervention (control)

Participants allocated to this group will receive three exercises as described by van Dieën et al. [49]. These exercises are commonly prescribed for patients with chronic low back disorders. The exercises are knee to chest, lumbar rotation, and bridging. The description and dosage of the exercise are provided in Table 2.

**Table 2:** Minimal intervention

	Exercise type	Description	Dosage
1.	Knee to chest	In crook lying position, pull both knees to the chest with interlocked fingers until a comfortable stretch is felt in the hip and lower back. Maintain the highest position	5–10 s hold, 10 reps
2.	Lumbar rotation	In crook lying position, slowly rock knees from side to side as far away as possible and maintain each position	5–10 s hold, 10 reps
3.	Bridging	In crook lying position, lift the pelvis in a straight line as far up as possible and maintain the highest position	5–10 s hold, 10 reps

### Adverse events and safety

Serious adverse events (AEs) are generally rare with therapeutic exercise interventions. All participants will be informed before enrollment about the possibility of experiencing some common AEs associated with exercises such as mild muscle or joint pain and muscle pull, which are often self-limiting. AEs will be recorded during each treatment session as part of the data collection. However, in case of any serious adverse experience (e.g., exacerbating joint pain, marked joint swelling, light-headedness, angina, and shortness of breath or dizziness), it will be promptly reported to the clinical authorities for physician evaluation and further action. Additionally, reports will be sent to the Health Research Ethics Committee of National Orthopedic Hospital, Dala, Kano State, Nigeria. The participants will be allowed or be asked to withdraw from the study if they make such a request or develop serious AEs.

### Sample size calculations

Considering the minimum detectable change of 1.0 cm or 100 mm reported for LMM CSA [50], the sample size was estimated a priori to detect a minimum difference of 1.0 point  in LMM CSA between LSER and LSE, or 2.0 points between LSER and minimal intervention, assuming a common standard deviation (*SD*) of 2.5 points based on our pilot study [29], a medium effect size of 0.32, an alpha of 0.05, statistical power of 85%, and a correlation of 0.5 among repeated measures,  for between–within repeated measures analysis of variance (ANOVA). The calculations revealed that a sample size of 75 is required. However, while anticipating a 20% dropout rate (*n* = 15), a total sample size of 90 (30 per group) will be finally required. The sample size was calculated using G Power 3.1.9.2 software [51].

### Data processing and statistical analyses

All data will be carefully recorded in a logbook and electronically using Microsoft Excel sheets. Data values will be double-checked for missing values and errors before transfer into SPSS for reporting and statistical analysis. Intention to treat (ITT) analysis will be applied with all randomized participants who have any outcome data available for analysis, included in the trial regardless of the presence or absence of follow-up. All statistical analyses will be conducted on IBM SPSS Statistics version 23.0 (IBM Co., Armonk, NY, USA) with a *P*-value < 0.05 to be considered significant for all tests.

A normality test for the dependent variables will be examined using the Kol mogorov–Smirnov and Shapiro–Wilk test. Descriptive analysis will be used to summarize the data with the use of mean (SD), frequencies, and percentages. One-way ANOVA (for normally distributed data) or Kruskal–Wallis (for skewed data) test will be used to compare baseline continuous variables whereas the chi-square test will be used to compare baseline categorical variables among the three groups. Mixed between–within subject ANOVA will be applied to evaluate time effect, group effect, and group by time interaction effect  in all outcomes if the data is normally distributed. Otherwise, Friedman’s ANOVA and Kruskal–Wallis test will be applied to evaluate within-group and between-group differences, respectively, when the data is skewed. Post hoc analysis will be applied for any significant within- or between-group differences detected. The effect size will be computed to evaluate the magnitude of change in all the outcomes. To examine the relationship between changes in LMM CSA and pain or disability, correlation and regression analyses will be performed for all the intervention groups.

### Trial steering committee

The trial steering committee is formed by the Research Ethics Committee of National Orthopedic Hospital, Dala, Kano State, Nigeria. The committee by liaising with the principal investigator is responsible for the continuation or discontinuation of the trial, ensuring that the study protocol is adhered to, and for study protocol amendments, if necessary. The steering committee will meet at least once a month to discuss the study progress.

### Trial audit

The trial steering and ethics committees will meet twice per month to review the conduct of the study throughout the trial period.

### Dissemination

The results of this study will be disseminated through publications in peer-reviewed journals and also presented at national or international conferences, regardless of whether the results are positive, negative, or inconclusive.

### Trial amendments

Any amendment to the study protocol will be reported to the Health Research Ethics Committee of National Orthopedic Hospital, Dala, Kano State, Nigeria, as well as updated in the Pan African Clinical Trials Registry.

## Discussion

Previous small RCT (*n* = 41) suggests that a 4-week LSE training with RUSI biofeedback may improve LMM CSA in patients with non-specific acute LBP compared to analgesics alone [28]. In contrast, in an RCT by Partner and colleagues [31] to compare LMM thickness in patients with NCLBP before and after a single session of LSE with and without biofeedback including the use of RUSI, no significant inter-group difference was detected for LMM thickness. Their study [31] however is underpowered, suffered a small sample size (*n* = 22), and included a single exercise session which may not be adequate to induce a significant change in the outcome observed. Taking into account  methodological limitations of these trials [28, 31], a powered RCT will be conducted to determine  the effectiveness of 8-week LSE training with RUSI biofeedback compared to LSE training without biofeedback, or minimal intervention in individuals with NCLBP. After the intervention, outcomes will be also evaluated at the 3-month follow-up and possibly at the 6-month or 12-month follow-up. If our hypothesis is correct, this study has the potential to inform  the decision to change practice by providing evidence for the clinical effectiveness of LSE with RUSI biofeedback on enhancing recovery of LMM size, which is commonly implicated in LBP and reoccurrence [5]. 

As a secondary objective, the present study will seek to examine the relationship between p ost-treatment change in LMM CSA and pain as well as disability. This may help to establish causality effect regarding LSE training with RUSI biofeedback on LM M morphology (i.e., CSA) and LBP and LBP-related disability.

This study however is without some limitations. The measurement of LMM CSA will be only performed at the L5 level even though aberrations in LMM morphology are commonly observed at this level [30, 41], besides evidence of recovery following specific rehabilitation [28, 30]. Therefore, any changes in the LMM CSA in other levels of the lumbar spine following rehabilitation could not be detected. Furthermore,  other important LMM morphological-related outcomes such as fat infiltration, volume, and percentage thickness will not be evaluated due to limited resources. Nevertheless, the planned powered, single (assessor) blind, three-arm (including comparator and active control groups), parallel RCT design is the strength of our study.  

### Trial status 

The initial version of the study protocol (version 1.2) was approved on J anuary 16, 2018. The current version of the study protocol (version 1.4) was approved on  October 4, 2021. The first enrollment was on August 01, 2019. Follow-up is expected to be completed by September 30, 2021.

### Trial registration

The study trial was registered prospectively at the Pan African Clinical Trials Registry (https://pactr.samrc.ac.za/) on January 16, 2018 (registration number: PACTR201801002980602).

## Supplementary Information

**Additional file 1.** SPIRIT 2013 Checklist.

## Data Availability

After the study is completed, the data will be made available on request from the corresponding author.

## Abbreviations

AEs: Adverse events
ANOVA: Analysis of variance
CSA: Cross-sectional area
ITT: Intention to treat
LBP: Low back pain
LMM: Lumbar multifidus muscles
LSE: Lumbar stabilization exercises
LSER: Lumbar stabilization exercises with real-time ultrasound imaging biofeedback
NCLBP: Non-specific chronic low back pain
NPRS: Numerical pain rating scale
MCS-12: Mental component scores
PCS-12: Physical component scores
QOL: Quality of life
RCT: Randomized controlled trial
RMDQ: Roland–Morris disability questionnaire
RUSI: Real-time ultrasound imaging
SD: Standard deviation
SPIRIT: Standard protocol items: recommendations for interventional trials
TrA: Transversus abdominis
